# Postinfectious COVID-19 Catatonia: A Report of Two Cases

**DOI:** 10.3389/fpsyt.2021.696347

**Published:** 2021-07-26

**Authors:** Tyler Torrico, Timothy Kiong, Carlos D'Assumpcao, Uyi Aisueni, Fouad Jaber, Katayoun Sabetian, Mohammed Molla, Rasha Kuran, Arash Heidari

**Affiliations:** ^1^Department of Psychiatry, University of California Los Angeles (UCLA)-Kern Medical Center, Bakersfield, CA, United States; ^2^Department of Internal Medicine, University of California Los Angeles (UCLA)-Kern Medical Center, Bakersfield, CA, United States; ^3^Department of Medical Education, Kern Medical Center, Bakersfield, CA, United States

**Keywords:** neuropsychiatry, neurobehavioral, SARS-CoV-2, COVID-19 outbreak, infectious disease

## Abstract

Neuropsychiatric symptoms are a common complication of COVID-19, with symptoms documented both during acute COVID-19 infection (parainfectious) and persisting or developing after the resolution of respiratory symptoms (postinfectious). Patients have presented with a variety of symptoms such as anosmia, thrombotic events, seizures, cognitive and attention deficits, new-onset anxiety, depression, psychosis, and rarely catatonia. Etiology appears to be related to disruption of regular neurotransmission and hypoxic injury secondary to systemic inflammation and cytokine storm. Although rare, catatonia and each of its subtypes have now been reported as complications of COVID-19 and therefore should be considered known to occur in both the parainfectious and postinfectious states. Diagnosis of catatonia in the context of COVID-19 should be considered when work-up for more common medical causes of encephalopathy are negative, there is no identifiable psychiatric etiology for catatonia, and there is a positive response to benzodiazepines.

## Introduction

Neuropsychiatric symptoms are a common complication of COVID-19. Patients have presented with symptoms such as anosmia, thrombotic events, seizures, cognitive and attention deficits, new-onset anxiety, depression, or psychosis (1). Neuropsychiatric complications have been documented both during acute COVID-19 infection (parainfectious) and persisting or developing after resolution of respiratory symptoms (postinfectious), with triggers for symptom onset remaining unclear (1–11). Understanding of the etiology for neuropsychiatric symptoms has been rapidly developing, with cerebrospinal fluid (CSF) findings (2, 3), magnetic resonance imaging (MRI) findings (3, 12), and electroencephalogram (EEG) findings (13) inconsistent for diagnostic purposes across different symptomatology (14). However, pathogenesis appears related to nervous system damage secondary to systemic inflammation and cytokine storm (IL-18, IL-6, IL-10, and TNF-alpha) (1).

Catatonia is a complex neurobehavioral condition whose pathophysiology remains unclear (15). The evidence for acute-phase activation (including cytokines and acute-phase proteins) in catatonia pathogenesis is sparse and conflicting (16). There are multiple reports describing all subtypes of catatonia occurring presumably secondary to COVID-19 in the absence of identifiable psychiatric etiology. Retarded catatonia (9) and malignant catatonia (10) have been reported as parainfectious complications, and excited catatonia was recently described as a postinfectious complication (11). In this report, we present two additional cases of postinfectious catatonia as complications of COVID-19.

## Case Report 1

A 36-year-old African American female with a past medical history of hypertension, type 1 diabetes, and incidental pancreatic head mass (6.7 cm × 5.4 cm, found 4 months prior during hospitalization for primary cesarean section), presented to the emergency department from a homeless shelter with subjective weakness, nausea, and slurred speech. She stated that she had a cough and fever about 2 weeks prior to admission but denied them on presentation. Vital signs were normal and required no supplemental oxygen, physical examination revealed dysarthric speech, unsteady gait, mild dysmetria. COVID-19 RNA was detected by reverse transcription polymerase chain reaction (RT-PCR) nasopharyngeal swab. C-reactive protein was undetectable. Erythrocyte sedimentation rate was 22 mm/h. Anti-nuclear antibody screen was negative. Urine toxicology was negative and admission labs were otherwise unremarkable. Computed tomography (CT) of the head and brain was negative for acute intracranial abnormalities.

Over the following 3 days, the patient became progressively drowsier and irritable; yelling at staff, which the patient's family noted was abnormal behavior. Her weakness progressed rendering her unable to move from bed to chair on her own and began refusing utensils, eating only by hand. The patient appeared catatonic by day 6, with immobility, mutism, refusal to eat most meals, and staring behaviors.

Lumbar puncture revealed normal opening pressure, mild lymphocytic pleocytosis (white blood cells 6/μL), normoglycorrhachia, and elevated protein level (103 g/dL). CSF did not grow any bacteria or fungus. The following CSF infectious serologies returned negative: Coccidioidomycosis (highly endemic region), West Nile IgM and IgG, Enterovirus PCR, herpes simplex virus (HSV) 1 IgG, HSV 2 IgG, California encephalitis virus IgG and IgM, western equine encephalitis IgG and IgM. MRI with and without contrast of the lumbar spine and brain were normal. A repeat lumbar puncture was obtained the following day and results with normalized cell count (white blood cells 3 /uL) and normalized protein (41 g/dL). Intravenous (IV) methylprednisolone 1,000 mg daily for 5 days was initiated empirically for suspicion of autoimmune encephalitis.

No gross improvement was seen after the methylprednisolone treatment, however, the patient refused gait assessment and was selectively mute, continuing to lie in bed with her head covered, refusing all medical care. Catatonia was suspected and 2 mg lorazepam trial was initiated 3 days later with the first dose administered intramuscularly along with haloperidol 5 mg and diphenhydramine 50 mg due to the patient's severe agitation. Ten-minutes after the first dose of lorazepam, the patient sat up at the bedside and was seen calling her family members, she participated with vital signs and maintenance medical management for the first time in over a week. A 1-h electroencephalogram was now allowed by the patient which showed no seizure activity and background slowing of 6–7 Hz. The patient's behavior continued to improve. Extensive psychiatric evaluation and the patient's family confirming no prior psychiatric history or episodes ruled out psychiatric origin of the patient's catatonia. The patient's ability to walk was significantly improved but still requiring a front-wheel walker. Prior to discharge, a third LP found normal CSF. CSF analysis was sent to Quest diagnostics NMDA-receptor antibodies and anti-Hu antibodies which returned negative. CSF COVID-19 PCR returned negative. Outpatient follow-up 1 week later showed further continued improvement no longer requiring the use of a front-wheel walker.

## Case Report 2

A 64-year-old Caucasian female with a past medical history of Roux-en-Y gastric bypass surgery, hypertension, and unspecified bipolar disorder was brought into the emergency department for altered mental status. History was obtained from the husband at the bedside as patient was selectively mute to the treatment team. The patient was in her normal baseline state of health (driving independently, appropriate social interactions) until ~2 months prior to this presentation. Her symptoms include waxing and waning mentation problems, selective mutism, minimal engagement with others and no longer pursuing her enjoyable activities. Instead, staring blankly most of the day and refusing to eat or drink consistently, which resulted in a 20-pound weight loss. The patient had one episode of hypomania over 10 years ago. Her home psychotropics include paroxetine 30 mg, and quetiapine 200 mg. The family denied other episodes of psychosis, mania, or suicidality in the patient.

The patient had a series of evaluations at an outside hospital for the same symptoms prior to presenting to our hospital. Six weeks prior to the current presentation, COVID-19 RNA was detected by RT-PCR nasopharyngeal swab despite her having no pulmonary symptoms and requiring no supplemental oxygen. She was advised over-the-counter zinc supplementation as an outpatient. At that time, MRI brain, 1-h EEG, and CSF showed no abnormalities. She reportedly returned to baseline and was discharged. She returned to the outside hospital 2 weeks prior to the current presentation for worsened altered mental status. At that time, she had a blood pressure of 190/101, all extremities flaccid, barely responsive to rub, eyes closed, making incomprehensible sounds. Her trachea was intubated for airway protection. She was given lorazepam 2 mg IV for suspected seizure. Shortly thereafter she hand wrote that she would like to be extubated. She was successfully extubated, entirely alert and oriented, and discharged home. For ~1 week she was independent with all her activities of daily living. However, she developed episodes of urinary and fecal incontinence, along with worsening selective mutism which led to the current presentation.

Mental status on admission revealed drowsiness, but with significant prompting, she is oriented to month and year. Her strength was fully intact in the upper and lower extremities bilaterally. Deep tendon reflexes were brisk throughout. She had decreased sensory response to pinprick in both feet and decreased position sense to both lower extremities. No dysdiadochokinesia was found, and the Babinski sign was negative. Patient was administered 2 mg lorazepam trial. Shortly after, she was appropriately engaging in conversation but unoriented to the situation. She denied recent depression, psychotic symptoms, obsessive symptoms, posttraumatic stress disorder symptoms. However, as the interview progressed patient speech became increasingly pressured and she began making bizarre statements and laughing inappropriately. She attempted to stand but required assistance and was noted to have an unsteady gait, requiring assistance with ambulation. The patient's mentation returned to selectively mute and confused approximately an hour later.

Twenty-four-hour EEG was normal with post dominant rhythm of 8–9 Hz, no seizures, and no postictal activity. MRI of the brain was normal. MRI of the lumbar spine showed multilevel degenerative change most pronounced in the lumbar spine and there was a mild spinal canal and moderate to severe bilateral neuroforaminal narrowing without cord compression. Serum copper level was 11 mcg/dL (normal 70–175 mcg/dL). Serum ceruloplasmin was 6 mg/dL (normal 18–53 mg/dL) and serum zinc was near the upper limit of normal at 126 mcg/dl (normal 60–130 mcg/dL). Copper 2 mg daily replacement was initiated intravenously for zinc-induced copper deficiency myeloneuropathy. Serum B12 levels were elevated. Serum folate was normal but folate storage in RBC was not measured. LP found an opening pressure of 7 cm of H_2_O. CSF analysis revealed traumatic tap, mild lymphocytic predominant pleocytosis (white blood cells 12/μL), normoglycorrhachia, and normal protein level. CSF cytology was normal. Additionally, CSF IgG synthesis rate and IgG oligoclonal bands were within normal limits. CSF HSV 1/2, West Nile, Cocci, Enterococcus, and COVID-19 PCR all returned negative. University of Pennsylvania autoimmune encephalitis evaluation CSF panel (Anti-NMDA receptor, Anti-LGI1, Anti-GAD65, Anti GABA-B, Anti-CASPR2, Anti AMPA-R) returned negative. Human immunodeficiency virus antibody/antigen 4th generation testing, syphilis antibody ELISA testing, and CSF acid-fast bacilli microscopy and culture were negative. Antinuclear antibodies, Sjogren antibodies (SS-A, SS-B), Ribosomal P antibody returned negative. Morning cortisol levels were normal.

The patient was transferred to the intensive care unit for close observation and airway monitoring as she was found to be obtunded. At this time, she was only minimally able to nod/shake her head in response to questions. Minimally able to move her upper and lower extremities, able to weakly grasp her fingers when prompted. The patient underwent a CT scan of the abdomen to rule out occult malignancy which revealed a polypoid mass in the periampullary region spanning 1.8 cm. Esophagogastroduodenoscopy was deferred at that time due to specialized equipment needed to examine the ampulla in a Roux-en-Y patient. On day 5 IV methylprednisolone 1,000 mg per day was initiated for 7 days empirically for autoimmune or paraneoplastic encephalitis.

After no gross improvement to IV methylprednisolone treatment, a lorazepam trial was re-initiated starting at lorazepam 0.5 mg three times daily. Over the next 3 days, she started to open her eyes when her name was called. The patient started to speak with a weak tone and endorsed feeling fatigued. Over the next week, the patient's symptoms continued to improve with increasing doses of clonazepam, which was chosen for its longer half-life. She became alert and oriented to name, place, month, and day. The patient was non-ambulatory and required a wheelchair and assistance with transfers. After 29 days in the hospital, she was discharged to a skilled nursing facility to assist with her physical therapy needs on oral clonazepam and oral copper replacement.

## Discussion

Postinfectious COVID-19 catatonia is a rare neuropsychiatric complication of COVID-19. Zain et al. (11) recently described excited type catatonia as a delayed complication of COVID-19. However, the term postinfectious may be more suitable to describe this phenomenon as parainfectious catatonia has also been rarely reported (9, 10). Additionally, this is consistent with the general nomenclature of other timing-associated complications of infectious diseases (1, 17). Catatonia is a behavioral syndrome marked by an inability to move normally which is classically associated with psychiatric conditions but it also an underrecognized cause of altered mental status in medically ill (18). In this report, administration of lorazepam resulted in rapid resolution of both patient's motor symptoms with improved cognition, strongly suggest catatonia.

**Figure 1:**
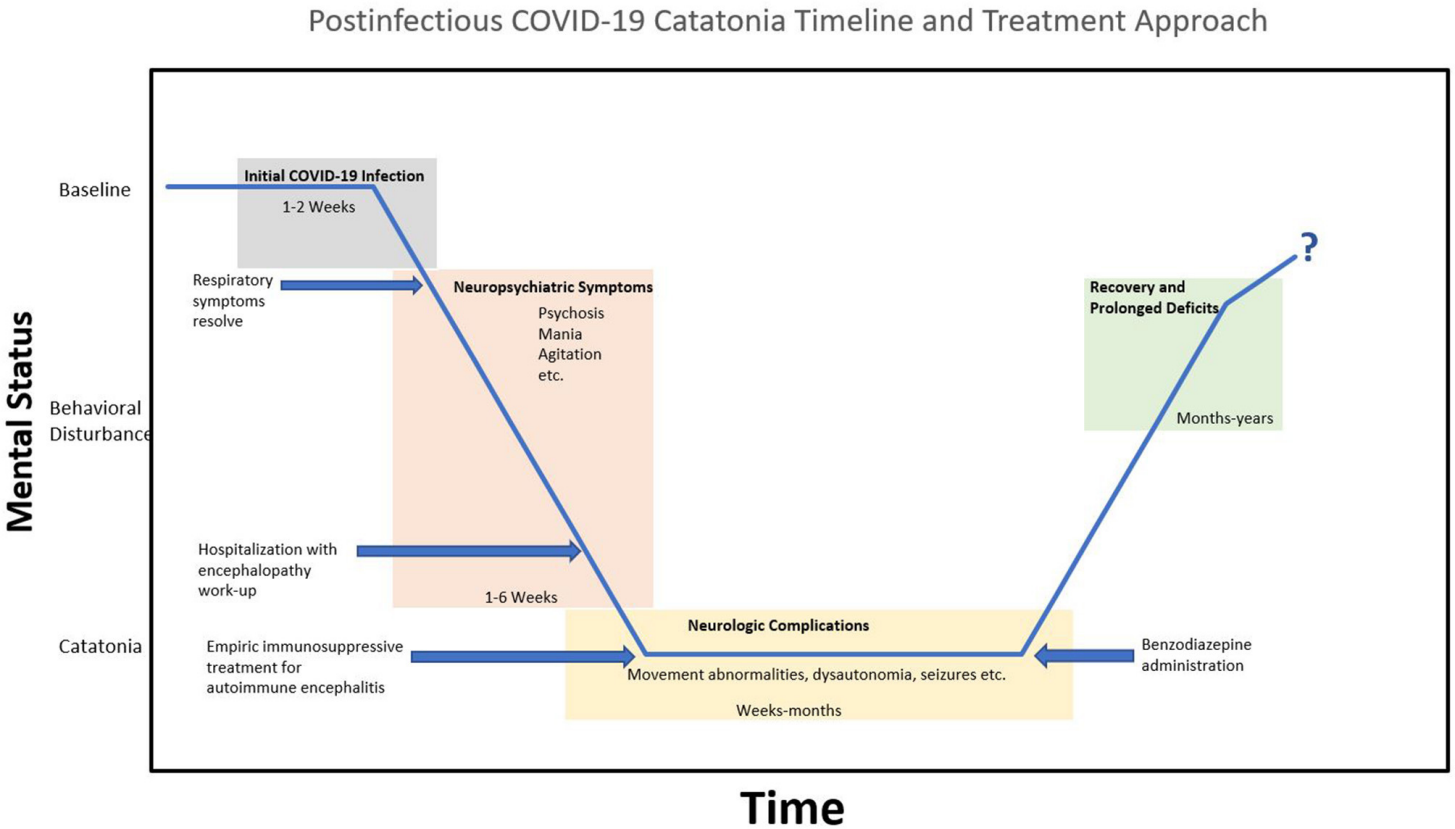
Generalized timeline of postinfectious COVID-19 catatonia and treatment approach. Initial COVID-19 infection is followed by the onset of neuropsychiatric symptoms. Hospitalization results in a typical encephalopathy work-up, revealing non-specific findings on MRI brain, EEG, and CSF findings; leading to suspicion of autoimmune encephalitis. Symptoms generally worsen with movement abnormalities and dysautonomia has been reported (i.e., malignant catatonia). With no clinical improvement after empiric immunosuppressive therapy, benzodiazepines are administered. This initiates the recovery phase, but prognosis and prolonged deficits are unknown at this time.

It appears that in most cases of COVID-19 associated neuropsychiatric symptoms the etiology is related to systemic inflammation and cytokine storm. Cytokine elevation and microglia activation result in increased kynurenine, quinolinic acid, glutamate, resulting in depletion of neurotransmitters and increased coagulation. Altered neurotransmission and hypoxic injury lead to neuronal dysfunction and loss. Symptomatically, it is likely that symptoms differ depending on the brain areas involved (1). Timeline-wise, there is inconsistency for postinfectious neuropsychiatric complications. However, a generalized timeline for patients with catatonia after COVID-19 is displayed in Figure 1.

In case 1, EEG was unable to be obtained before lorazepam administration and is therefore largely confounded by benzodiazepine administration. Therefore, it is possible that nonconvulsive status epilepticus (NCSE) was the pathogenesis for the patient's negative phenomenology including catatonia. Although no positive phenomenology of a seizure disorder was observed, they are often overlooked in NCSE. Case 2 had 1 h EEG screening 6 weeks before presentation without evidence of NCSE, and 24 h EEG this hospitalization, also without evidence of NCSE. Regardless, symptomatic treatment for catatonia and NCSE is lorazepam. Although patient 2 in this report had mentation returned to baseline, she was unable to walk prior to discharge because she still suffered from zinc-induced copper deficiency myeloneuropathy as her history of Roux-en-Y gastric bypass placed her at high risk for micronutrient deficiencies, which was exacerbated by zinc supplementation after initial COVID-19 diagnosis. Copper deficiency myeloneuropathy is not known to be associated with catatonia.

New-onset neuropsychiatric symptoms associated with COVID-19 raise concern for viral invasion with some clinicians reporting encephalitis. The understanding of encephalitis associated with COVID-19 is still developing but usually occurs in patients who are critically ill with COVID-19 infection. Postinfectious COVID-19 encephalopathy, i.e., a prolonged confusional state in the absence of hypoxia, is rare (2, 19). COVID-19 encephalopathy is not reported to have a positive clinical response to benzodiazepine therapy, and EEG findings are largely non-specific and inconclusive (3, 5). Additionally, cases of COVID-19 encephalopathy have usually had either abnormal MRI findings (not found in both cases presented), with or without CSF findings such as SARS-CoV-2 spike 1 and spike 2 protein, nucleoprotein antigens (4), COVID-19 RNA detected by RT-PCR in the CSF (5) (which patient 1 tested negative for).

The postinfectious state of COVID-19 with new-onset neuropsychiatric symptoms in the context of negative MRI findings, non-specific EEG findings, and negative CSF, raises suspicion for undetected autoimmune encephalitis due to clinical similarities (20). Anti-NMDA receptor encephalitis is the most common cause of autoimmune catatonia (16). Interestingly, anti-NMDA receptor encephalitis has been rarely diagnosed associated with COVID-19 (6–8). In this report, patient 1 tested negative for antibodies against NMDA and Hu proteins, and patient 2 tested negative for antibodies against NMDA, LGI1, GAD65, GABA-B, CASPR2, AMPA-R antibodies. Further, the general treatment for autoimmune encephalopathy is immunosuppression, and in this report, neither patient appeared to have a positive clinical response to empiric IV high dose methylprednisolone. However, both patients in this report had pancreatic masses, and although paraneoplastic encephalopathy can be less responsive to immunosuppression, pancreatic masses are rarely associated with paraneoplastic syndromes and therefore considered an unlikely etiology (21).

In conclusion, neuropsychiatric symptomatology is broad in postinfectious COVID-19. As understanding of the pathogenesis remains fairly limited, symptomatic management is an appropriate strategy. Although rare, catatonia is now documented multiple times as a complication of COVID-19 and should be considered a known potential complication in both the parainfectious and postinfectious state. Diagnosis of catatonia in the context of COVID-19 should be considered when work-up for more common medical causes of encephalopathy are negative, there is no identifiable psychiatric etiology for catatonia, and there is a positive response to benzodiazepines.

## Data Availability Statement

The data displayed during the current study are not publicly available as they are part of protected health information of the patients described. The data are however are available upon reasonable request and after approval. Requests to access the datasets should be directed to tylertorrico@kernmedical.com.

## Ethics Statement

The studies involving human participants were reviewed and approved by Kern Medical Center Institutional Review Board. The patients/participants provided their written informed consent to participate in this study. The patients provided written, informed consent for the publication of these case reports (including all data and images).

## Author Contributions

All authors listed have made a substantial, direct and intellectual contribution to the work, and approved it for publication.

## Conflict of Interest

The authors declare that the research was conducted in the absence of any commercial or financial relationships that could be construed as a potential conflict of interest.

## Publisher's Note

All claims expressed in this article are solely those of the authors and do not necessarily represent those of their affiliated organizations, or those of the publisher, the editors and the reviewers. Any product that may be evaluated in this article, or claim that may be made by its manufacturer, is not guaranteed or endorsed by the publisher.
